# Surgical Outcomes of Central Airway Adenoid Cystic Carcinoma: A Retrospective Single-Center Analysis

**DOI:** 10.5761/atcs.oa.25-00210

**Published:** 2026-02-18

**Authors:** Toshihiko Soma, Shinjiro Nagai, Takashi Indo, Satoshi Ueda, Naoko Imanishi, Mitsuhiro Ueda, Yoshihiro Miyamoto

**Affiliations:** Department of Thoracic Surgery, National Hospital Organization Himeji Medical Center, Himeji, Hyogo, Japan

**Keywords:** adenoid cystic carcinoma, adjuvant therapy, airway reconstruction, central airway tumor, long-term outcomes

## Abstract

**Purpose:**

Central airway adenoid cystic carcinoma (CAACC) is a rare malignancy lacking a standard treatment approach and often precluding complete resection. This study assessed the surgical outcomes of patients with CAACC treated at a single institution.

**Methods:**

We retrospectively reviewed patients who underwent surgical resection for CAACC between September 2013 and August 2021.

**Results:**

Eight patients (mean age: 51.5 years) were included. Tumor locations were bronchus (*n* = 1), trachea (*n* = 4), carina and bronchus (*n* = 2), and carina and trachea (*n* = 1). Surgical procedures included sleeve lobectomy (*n* = 1), tracheal resection (*n* = 4), sleeve pneumonectomy (*n* = 2), and carinal resection with reconstruction (*n* = 1). Preoperative radiation and bronchoscopic tumor resection were performed in 1 patient each. One patient died from a postoperative tracheoinnominate artery fistula. Major complications included recurrent laryngeal nerve palsy (*n* = 3). Adjuvant therapy was provided for positive or uncertain margins. During a median follow-up of 6 years, 2 patients developed recurrence but remained alive at the last follow-up. The 5-year overall survival rate was 72.9%.

**Conclusion:**

Surgical resection with airway reconstruction and adjuvant therapy can offer long-term disease control in CAACC, though life-threatening complications warrant careful consideration.

## Introduction

Central airway adenoid cystic carcinoma (CAACC) is a rare malignancy characterized by slow, indolent growth and often presents with nonspecific or minimal symptoms, contributing to frequent misdiagnosis as bronchial asthma and delayed diagnosis in many cases.^1,2)^ In some instances, this delay can result in acute airway obstruction.^3,4)^

Surgical resection remains the cornerstone of treatment and has been associated with improved survival outcomes.^1,5,6)^ However, complete resection is frequently challenging because of the tumor’s tendency for submucosal and perineural infiltration along the airway. Reported perioperative mortality rates vary widely, ranging from 1% to 25%,^7–10)^ underscoring the technical difficulty and risks involved.

Adjuvant radiotherapy (RT) is commonly recommended in cases with microscopically positive surgical margins (R1 resections).^7,11,12)^ Nevertheless, its efficacy remains uncertain due to the lack of randomized controlled trials assessing its therapeutic value.^7,10,13–16)^

Because of the rarity of CAACC, clinical evidence remains limited, and standardized treatment protocols have yet to be established.^1)^ Continued accumulation and analysis of clinical cases are necessary to refine treatment strategies and improve patient outcomes.

This study retrospectively evaluated the surgical outcomes and postoperative complications in patients with CAACC treated at our institution, with the aim of contributing further clinical insight into this rare but clinically significant disease.

## Patients and Methods

This study was approved by the Institutional Review Board of the National Hospital Organization Himeji Medical Center (Institutional Review Board No. 2025-5; approval date: April 11, 2025). The requirement for informed consent was waived due to the retrospective nature of the study and the use of anonymized data.

A retrospective analysis was conducted on patients with localized CAACC who underwent surgical resection at our institution between September 2013 and August 2021. Survival outcomes were analyzed using the Kaplan–Meier method to estimate the 5-year overall survival rate. All statistical analyses were performed using EZR (version 4.3.1; Saitama Medical Center, Jichi Medical University, Saitama, Japan), a graphical user interface for R (The R Foundation for Statistical Computing, Vienna, Austria).

Preoperative interventions were implemented in patients with imminent airway compromise due to central airway obstruction to secure airway patency. The surgical approach was selected based on tumor location and included cervical collar incision, median sternotomy, or intercostal thoracotomy.

To reduce tension at the anastomotic site, the cartilaginous portion of the intrathoracic trachea was circumferentially mobilized, while preserving the membranous portion to maintain adequate vascular perfusion. During anastomosis, tracheal ends were approximated using traction sutures, followed by definitive suturing. When additional tension relief was necessary, a suprahyoid release, pulmonary ligament dissection, or caudal pericardial incision was performed. A pericardial fat pad or omental flap was applied over the anastomotic site in select cases to enhance healing and provide tissue support. Postoperative chin-to-chest suturing was not performed in any of the cases.

Bronchotracheal anastomosis was performed using interrupted 3-0 absorbable braided synthetic sutures, with 13–15 stitches typically required. Intraoperative frozen section analysis was utilized to assess resection margins. In cases where microscopic tumor involvement was identified and further resection was unfeasible, the resection was classified as margin-positive (R1). No cases of grossly positive margin (R2) resection were recorded. Postoperative endoscopic evaluation of tracheal and bronchial anastomoses was routinely conducted within 1–2 months following surgery.

During the preparation of this work, the authors used ChatGPT to check for grammatical errors. After using this tool, the authors reviewed and edited the content as needed and take full responsibility for the content of the publication.

## Results

**Fig. 1:**
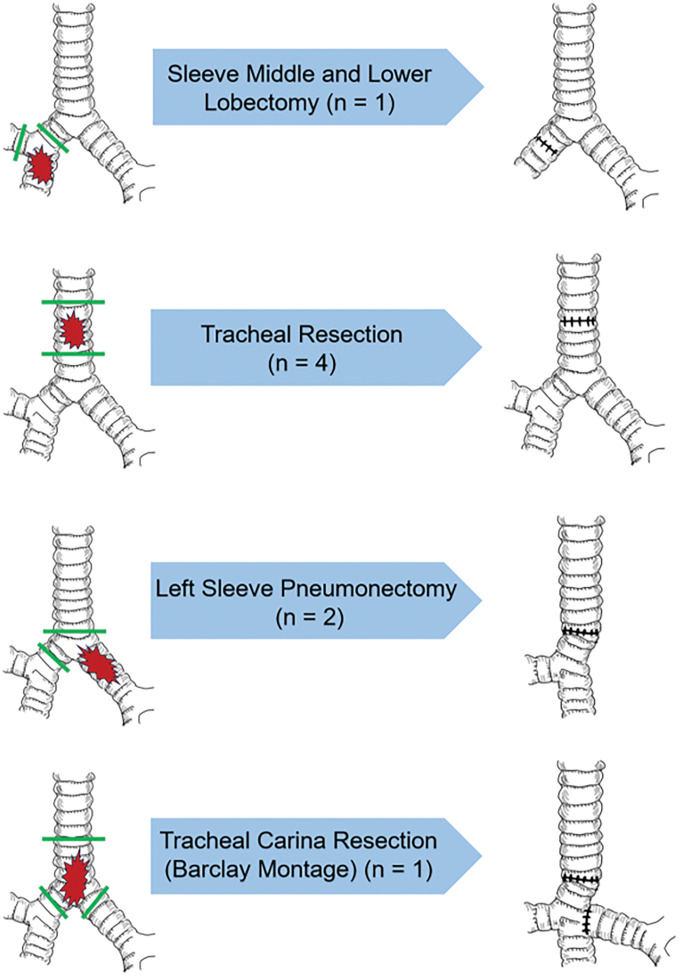
Tumor locations and resection methods. Anatomical locations of the tumors and the corresponding surgical resection methods used.

A total of 8 patients (5 men and 3 women) with localized CAACC were included. The mean age was 51.5 years (median: 46 years; range: 33–83 years). Tumor involvement was confined to the bronchus (*n* = 1), trachea (*n* = 4), carina and bronchus (*n* = 2), and carina and trachea (*n* = 1). **Figure 1** summarizes the tumor locations and the corresponding resection methods. A summary of patient demographics, preoperative interventions, surgical procedures, tumor characteristics, complications, postoperative course, and adjuvant therapies is provided in **Table 1**. Further details are described below; case numbers correspond to those in the table.

**Table 1:** Summary of surgical procedures, preoperative interventions, tumor characteristics, complications, postoperative course, and adjuvant therapies

	Case no.							
	1	2	3	4	5	6	7	8
Age/gender	83/M	33/M	38/M	38/M	70/M	41/F	56/F	53/F
Incision site	Right thoracotomy	Sternotomy	Cervical collar incision	Sternotomy	Sternotomy	Left thoracotomy	Left VATS, right thoracotomy	Right thoracotomy
Surgical procedure	Sleeve MLL	TR	TR	TR	TR	Left SP	Left SP	Tracheal carina resection (Barclay montage)
Preoperative intervention	None	None	None	70-Gy RT	None	None	None	Bronchoscopic intervention
Suprahyoid release	−	+	−	+	+	−	−	+
Coverage	Pericardial fat pad	—	—	—	—	—	—	Omentum
Tumor size	19 mm	>21 mm	22 mm	16 mm	8 mm	50 mm	50 mm	>41 mm
Margin	R1	R1	R0	R1	R0	R0	R1	R1
Complications (Grades I–IV)	–—	Atelectasis	—	Right RLNP	—	Left RLNP	—	Bilateral RLNP
Complications (Grade V)	—	—	—	TIF	—	—	—	—
LOS (days)	13	13	10	25	15	16	16	77
Adjuvant therapy	None	60-Gy RT	50-Gy RT	N/A	None	50-Gy IMRT + cisplatin/S1	50-Gy RT	60-Gy IMRT

M: male; F: female; VATS: video-assisted thoracoscopic surgery; MLL: middle and lower lobectomy; TR: tracheal resection; SP: sleeve pneumonectomy; RT: radiotherapy; R1: microscopically margin-positive resection; R0: negative margin resection; RLNP: recurrent laryngeal nerve palsy; TIF: tracheoinnominate artery fistula; LOS: length of stay; N/A: not applicable; IMRT: intensity-modulated radiation therapy

### Preoperative interventions

Two patients (Cases 4 and 8) required preoperative interventions due to impending respiratory distress from malignant central airway obstruction. In Case 4, the patient initially presented with a 64-mm tracheal tumor that posed a significant risk of airway obstruction. Therefore, RT was selected by the referring institution to achieve rapid tumor shrinkage and prevent possible asphyxiation. The patient declined a blood transfusion for religious reasons. Thus, tumor reduction was also considered beneficial for decreasing surgical invasiveness and perioperative blood loss. Accordingly, conventional external-beam RT at a total dose of 70 Gy was administered.

In Case 8, airway patency was restored via bronchoscopic tumor debulking using biopsy forceps and argon plasma coagulation, performed 7 weeks before surgery.

### Surgical procedures

One patient with bronchial involvement (Case 1) underwent sleeve resection of the right middle and lower lobes via a right thoracotomy. Tracheal resection and reconstruction were performed in 4 patients (Cases 2–5). Among the 3 patients with carinal involvement (Cases 6–8), Case 6 underwent sleeve left pneumonectomy via left thoracotomy alone, whereas Case 7 underwent the same procedure using a left video-assisted thoracoscopic surgery–assisted right thoracotomy approach. Case 8 underwent carinal resection and reconstruction.

In Case 8, resection of approximately 5 cm of the trachea, including the carina, was required. Transection of the pulmonary ligament and caudal pericardiotomy allowed elevation of the right hilar structures, enabling a tension-free anastomosis between the right main bronchus and the trachea. A subsequent end-to-side anastomosis was performed between the left main bronchus and the intermediate bronchial trunk using the Barclay-type carinal reconstruction technique.^13)^

In cases where significant anastomotic tension was anticipated (Cases 2, 4, 5, and 8), a suprahyoid release was performed. Anastomotic reinforcement was achieved using a pericardial fat pad (Case 1) or an omental flap (Case 8), when deemed necessary. Operative time ranged from 150 to 592 min (median: 266.5 min), and estimated blood loss ranged from 30 to 312 mL (median: 192.5 mL).

### Surgical mortality and complications

One patient (Case 4) died postoperatively due to a tracheoinnominate artery fistula that developed at the anastomotic site on postoperative day 27. No other anastomotic complications, such as tracheal stenosis, anastomotic fistula, or tracheomalacia, were observed.

Postoperative complications included recurrent laryngeal nerve paralysis (*n* = 3; Cases 4, 6, and 8) and atelectasis (*n* = 1; Case 2). In Case 4, unilateral recurrent laryngeal nerve paralysis resolved within days, while in Case 6, it resolved over several months. Case 8 experienced bilateral recurrent laryngeal nerve paralysis requiring tracheostomy, which was closed on postoperative day 69 following recovery of vocal cord function. The patient with atelectasis required bronchoscopic suctioning. Postoperative hospital stay ranged from 10 to 79 days (median: 15.5 days). None of the patients who underwent suprahyoid release developed swallowing dysfunction or aspiration.

### Postoperative therapy and prognosis

Five patients received postoperative RT: 3 received conventional external-beam RT and 2 received intensity-modulated radiation therapy (IMRT). One patient (Case 6) received combined chemoradiotherapy (cisplatin/S1 plus 50-Gy IMRT). Among the 5 patients with microscopically positive margins (R1), three (Cases 2, 7, and 8) underwent adjuvant RT 2 months postoperatively. Case 1, despite positive margins, did not receive adjuvant therapy due to advanced age (83 years) and chronic respiratory failure. Among the 3 patients with negative margins (Cases 3, 5, and 6), Case 6 received chemoradiotherapy because of concerns regarding close margins. Case 3, a younger patient with cervical tracheal involvement, received 50-Gy RT 6 months postoperatively. No late-onset anastomotic complications were observed during follow-up.

Among the 8 patients overall (R0 = 3, R1 = 5), recurrence occurred in 2 patients: Case 6 (R0) and Case 7 (R1). Case 6 developed distant metastasis in the right lung 3 years and 11 months postoperatively; the disease remained stable after treatment with ipilimumab/nivolumab, and the patient was alive at 6 years and 2 months postoperatively. Case 7 experienced local recurrence with left pleural dissemination and distant metastasis in the right lung 3.5 years postoperatively; the patient remained alive with disease at 11 years and 9 months postoperatively after multiple cycles of chemotherapy and RT. Recurrence occurred in both R0 and R1 patients, but notably, no recurrences were observed at the tracheal or bronchial anastomosis sites in any patient.

**Fig. 2:**
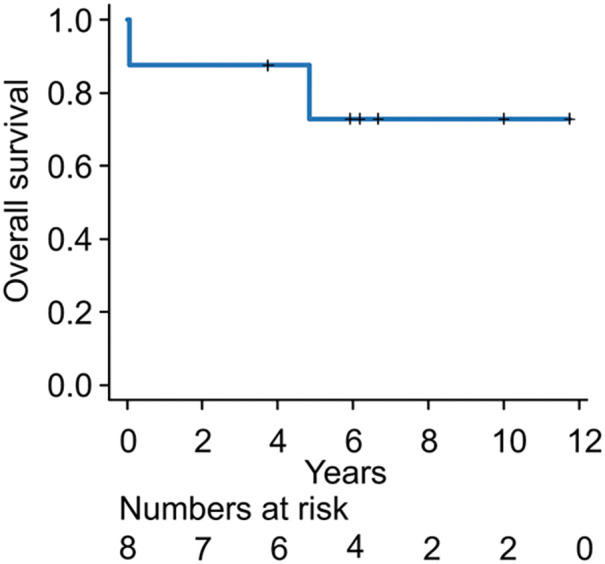
Kaplan–Meier survival curve after CAACC resection. The 5-year overall survival rate was 72.9%. Survival analysis was performed using the Kaplan–Meier method. CAACC: central airway adenoid cystic carcinoma

The follow-up period ranged from 27 days to 11 years and 9 months (median: 6 years and 1 month). **Figure 2** shows the Kaplan–Meier survival curves. The 5-year overall survival rate was approximately 72.9%.

## Discussion

CAACC is a rare malignancy that is frequently misdiagnosed as bronchial asthma due to its slow progression and nonspecific symptoms.^3,4)^ As a result, many patients remain asymptomatic until they present with advanced disease and significant airway obstruction. Various endoscopic interventions—such as laser therapy, bronchial stenting, and bronchoscopic tumor debridement—and radiation therapies, including external-beam radiation and high-dose brachytherapy, have been used to relieve airway obstruction.^17,18)^ However, these approaches generally lack curative potential and are associated with recurrent airway stenosis, often necessitating definitive surgical treatment.^19)^ Likewise, radiation therapy alone offers limited efficacy in cases of rapidly progressive airway compromise, making it suboptimal as a sole modality in such situations.^20)^

A review by Ran et al.^1)^ reported a 5-year overall survival rate of 86.4% for patients undergoing surgical resection alone, significantly higher than the 34.9% rate for those treated with radiation alone. Surgical resection, when feasible at the time of diagnosis, not only relieves airway obstruction but also offers a potential cure, highlighting its importance as a primary therapeutic strategy. However, immediate resection may not be viable due to a patient’s clinical status or logistical constraints. In such instances, initial airway stabilization via endoscopic or radiation therapy may serve as a bridge to definitive surgical treatment.

Nonetheless, preoperative radiation is known to impair tissue healing and vascularity, particularly in airway reconstruction.^21)^ Several previous studies have cautioned against administering high-dose radiation before tracheobronchial surgery because of the increased risk of anastomotic dehiscence, fistula formation, and impaired wound healing.^22,23)^ In our series, the only postoperative mortality occurred in Case 4, who underwent tracheal resection following high-dose preoperative RT (70 Gy). In this case, RT had initially been selected at the referring institution to secure airway patency and to reduce surgical risk in the context of blood transfusion refusal in a patient with a 64-mm tumor. Although the tumor size decreased to 4 cm, surgery was ultimately conducted because the residual lesion still posed a potential risk of airway compromise and the patient preferred definitive resection after understanding the possibility of persistent disease. This patient developed a tracheoinnominate artery fistula at the anastomotic site on postoperative day 27—a catastrophic complication that resulted in death.

We speculate that the high radiation dose adversely affected the microvascular environment around the trachea, rendering the anastomotic site more susceptible to tissue breakdown and fistula formation. Additionally, in this case, the anastomosis was adjacent to the innominate artery, where continuous arterial pulsation likely exerted mechanical stress on the irradiated, fragile tissue, further contributing to fistula development. In retrospect, interposing a well-vascularized tissue flap, such as the omentum or a muscle flap, might have mitigated the impaired wound healing caused by radiation and the mechanical stress from arterial pulsation, thereby reducing the overall risk of fistula formation.

Recurrent laryngeal nerve injury remains one of the most common postoperative complications due to the nerve’s anatomical course near the trachea.^7)^ In our study, 3 patients developed recurrent laryngeal nerve palsy: 2 cases of unilateral palsy resolved with conservative management (Cases 4 and 6), while 1 case of bilateral palsy required temporary tracheostomy (Case 8). These outcomes underscore the importance of careful intraoperative nerve preservation and prompt postoperative airway management.

In this series, intraoperative recurrent laryngeal nerve neuromonitoring (IONM) was not utilized. This was primarily because several procedures involved distal tracheal or carinal resections, where the recurrent laryngeal nerve is outside the main operative field and nerve preservation is not the immediate focus of airway reconstruction. However, given the incidence of nerve palsy observed in our cohort, the potential benefits of IONM should be considered. IONM provides real-time functional feedback during surgical manipulation and reduces nerve injury in thyroid and other neck surgeries. Similarly, its application may help identify early signs of nerve stress or impending injury during tracheal mobilization, particularly in cases requiring extensive dissection near the cervical or upper thoracic trachea or in patients with a previous history of radiation or inflammation. Therefore, incorporating IONM in cases selected in the future can be a useful adjunct to improve nerve preservation and reduce postoperative morbidity.

Incomplete resection margins are common in CAACC due to its infiltrative growth pattern. Previous reports indicate positive margins in 59%–63% of cases,^5,6,24)^ a finding mirrored in our cohort (62.5%). While intraoperative frozen section analysis can help guide margin assessment, the extent of resection is often limited by the risk of increased anastomotic tension and related complications.^5,7,8)^ When complete resection is not feasible, adjuvant RT has been associated with reduced local recurrence and improved survival outcomes.^7,11,12)^ Although its efficacy remains inconclusive due to the lack of randomized controlled trials,^10,14–16)^ our findings support its use. In our series, patients with positive margins received adjuvant RT or chemoradiotherapy, and none experienced local recurrence at the anastomotic site.

Overall, the 5-year survival rate was 72.9%, with recurrence observed in only 2 cases (Cases 6 and 7), both of whom remain alive with disease. These results suggest that long-term outcomes are favorable with appropriate multidisciplinary treatment. Furthermore, they highlight that surgical resection should aim for a balance between achieving oncological clearance and preserving safe anastomotic conditions. Excessive resection in pursuit of negative margins may lead to greater morbidity and should be avoided when it compromises patient safety.

## Conclusion

CAACC is a rare malignancy that often presents with life-threatening airway obstruction. Surgical resection remains the cornerstone of curative treatment, offering both immediate airway relief and the potential for long-term survival. Even in the presence of positive resection margins, multidisciplinary postoperative management—including adjuvant RT—can yield favorable outcomes. Ultimately, individualized treatment planning is essential, with careful consideration of the risks associated with potentially severe postoperative complications.

## Declarations

### Data availability

The clinical data used in this study are derived from electronic medical records and contain potentially identifiable patient information. Therefore, these data cannot be shared publicly for ethical and privacy reasons. The data will be shared on reasonable request to the corresponding author.
